# Role of Community Group Exposure in Reducing Sexually Transmitted Infection-Related Risk among Female Sex Workers in India

**DOI:** 10.1371/journal.pone.0078361

**Published:** 2013-10-24

**Authors:** Diwakar Yadav, Shreena Ramanathan, Prabuddhagopal Goswami, Lakshmi Ramakrishnan, Niranjan Saggurti, Shrabanti Sen, Bitra George, Ramesh Paranjape

**Affiliations:** 1 FHI 360, Green Park Extension, New Delhi, India; 2 Population Council, New Delhi, India; 3 National AIDS Research Institute, Bhosari, Pune, India; Midwestern University, United States of America

## Abstract

**Background:**

Empowering female sex workers (FSWs) to address structural barriers and forming community groups (CGs) through community mobilization are seen as essential components of HIV prevention programs in India. Taking the membership of a CG as an exposure intervention, we hypothesized whether participation in a CG lead to reduced sexually transmitted infections (STIs) and increased treatment-seeking behavior among FSWs in three selected states of India — Andhra Pradesh, Maharashtra and Tamil Nadu.

**Methods and Findings:**

The propensity score matching (PSM) approach examined the effect of CG membership, as against no membership, on STI-related risk, described as selected outcome measures — presence of any STI, self-reported STI symptoms, and treatment-seeking behavior among FSWs. A cross sectional bio-behavioral survey was administered in 2009–2010 and covered 7,806 FSWs through two-stage probability-based conventional and time location cluster sampling in 23 administrative districts of Andhra Pradesh, Maharashtra and Tamil Nadu. Only 2,939 FSWs were reported to be members of a CG and among them 4.5% had any STIs. A majority of FSWs were aged above 24 years (86.4%), had ever been married (73%), operated from a public place for solicitation (81.5%), and had ever received HIV test results (75.6%). The average effect of CG exposure was reduction in STI prevalence by 4%, while self-reported STI symptom treatment-seeking behavior increased by 13.7%.

**Conclusion:**

FSWs who were exposed to a CG were at a substantially lower risk of STIs than those who were unexposed. The FSWs exposed to a CG had a higher chance of seeking STI treatment from public and private health facilities. Collectivization related challenges must be overcome to provide access to tailored STI prevention and care services.

## Introduction

Formation of community groups (CGs) is a common output of community mobilization[1]. In HIV and AIDS programs, CGs and networks are seen as modes to strengthen demand for services, manage programmatic activities, and empower individuals and groups to reduce their vulnerability to HIV/sexually transmitted infection (STI) particularly among high-risk groups [2-4]. Apart from the delivery of public health services, community mobilization has also influenced many important HIV and AIDS policy decisions, and marginalized groups affected by HIV have found multiple benefits in coming together [5]. 

Commercial sex workers included in high-risk group population are highly vulnerable, disempowered and socioeconomically marginalized [6]. Understanding HIV/STI risk among female sex workers (FSWs) requires attention to both individual risk behaviors and the social-structural factors that shape the contexts of risk. Structural inequalities have led to an increased recognition of stigma reduction as a key component in HIV prevention and treatment [7-9] and justified the significance of community and structural factors in HIV/STI transmission [10,11].

Studies from developing countries have shown that community mobilization helps to empower sex workers, reduce vulnerability to HIV/STI and increase condom use [12-21]. In India, interventions targeting FSWs have been linked to increased knowledge of HIV/STI risk; consistent condom use with occasional and regular clients and non-client partners; and reduced STI prevalence [22-27]. Community mobilization of FSWs through the Sonagachi project in Kolkata, India, drew widespread interest [28], and growing literature suggests that Asian countries may continue to offer community mobilization as a structural approach to HIV and AIDS [29,30]. 

Avahan, the India AIDS Initiative was launched in 2003, and one of its major components is the formation of CGs through community mobilization as a strategy to empower FSWs to address structural barriers [31]. In addition to FSWs, Avahan supported interventions among other high-risk groups, such as clients of FSWs, men who have sex with men (MSM), transgender (TG) persons, injecting drug users (IDUs) and clients of sex workers in hotspots and through targeting long distance truck drivers in six high prevalence states of India [32]. Limited studies mentioned that community mobilization interventions help FSWs to use condoms and practice safe sex, access social entitlements, avail STI services from government health facilities and increase empowerment [30,33-41] . In the absence of evidence of lowered STI among members of CGs, this paper examines the association between exposure to a CG and presence of any STI, self-reported STIs and treatment-seeking behavior. In this paper, we briefly describe the FSWs characteristics among CGs membership and examine their impact on STI risk and treatment-seeking behavior among FSWs.

## Materials and Methods

### Ethical Statement

All participants in this study provided written informed consent. The integrated behavioral and biological assessment (IBBA) protocol and ethical clearances were obtained from Protection of Human Subjects Committee of FHI 360 and the Health Ministry Screening Committee of Indian Council of Medical Research (ICMR) institute and local ethical committees of the implementing institutes of ICMR [National AIDS Research Institute (NARI), Pune; National Institute of Medical Statistics (NIMS), New Delhi; National Institute of Nutrition (NIN), Hyderabad; and National Institute of Epidemiology (NIE), Chennai][42]. The IBBA dataset is available in public domain for research use through formal application process at NARI and also, survey instrument is available at IBBA website (www.ibbainfo.in). 

### Data, Study Setting and Sampling

The present study utilizes data from the second wave of the large-scale bio-behavioral cross sectional survey, known as IBBA, that was carried out in six high-prevalence states of India among FSWs and their clients, MSM, TGs, IDUs and truckers during 2009–2010. The IBBA is a significant component of Avahan’s evaluation strategy[43]. The collaborative efforts of many national-level organizations, such as ICMR-associated institutes including NARI, NIE, NIMS, NIN, and the Regional Medical Research Centre, Dibrugarh, and research agencies resulted in IBBA. FHI 360 (formerly Family Health International) was appointed the technical support agency to conduct the survey. IBBA provided data to measure the effect of program interventions on high-risk groups, in terms of behaviors as well as prevalence of HIV/ STI.

IBBA survey were conducted through face-to-face interviews and collection of blood and urine samples at cruising sites/settings to gather comprehensive behavioral and biological information on high-risk populations at the district level. Two-stage probability-based conventional and time location cluster sampling methods were used to sample 7,806 FSWs from brothel, home, lodged and street based settings [44]. We used data from 23 administrative districts in the three Indian states of Andhra Pradesh, Maharashtra and Tamil Nadu. 

### Outcome Variables

#### Presence of any Sexually Transmitted Infection

Presence of any STI was the first outcome variable measuring STI risk in blood and urine samples collected and tested in laboratory (biological examination). Syphilis was screened by the Rapid Plasma Regain (RPR) test and confirmed by *Treponema pallidum hemagglutination assay* (TPHA). Nucleic-acid amplification (Gen-Probe APTIMA Combo 2, Gen-Probe Inc., San Diego, California) tests were conducted to screen chlamydiasis (*C. trachomatis*) and gonorrhoea (*N. ganorrhoeae*). Participants who tested positive for any of the three STIs (syphilis, gonorrhoea and chlamydiasis) were categorized as ‘having any STIs or presence of STIs’.

#### Self-Reported any STI Symptoms

Self-reported any STI symptom was the second outcome variable measuring risk behavior among FSWs. Participants were asked if they had any STI symptoms in the past 12 months, for which the response options were: vaginal discharge, lower abdominal pain without diarrhea or menses, and genital ulcers or sores. We categorized the FSWs who reported any STI symptoms as ‘reporting STI symptoms’ and the remaining as ‘not reporting STI symptoms’. 

#### STI Treatment-Seeking Behavior

Treatment-seeking behavior for STIs was the third outcome variable measuring risk. Participants were asked if they had sought treatment/advice/medicine for any self-reported STI symptoms in the past 12 months, for which the response options were: sought advice/treatment/medicine from Avahan clinic, government clinic/hospital, non-governmental organization or charity-run clinic/hospital, and private clinic/hospital. We categorized the FSWs who reported seeking advice/treatment/medicine from clinic/hospital as ‘yes’ and those who did not as ‘no’.

#### Exposure to Community Group (CG)

Exposure to CG was defined as membership of a CG and meeting each other at least once a month. Participants were asked about their membership to CG (self-help group/community-based organization). This exposure variable was considered as indicator of community participation. 

### Explanatory Variables

Socio-demographic predictors such as age of respondents, formal schooling (read and write), marital status, source of income other than sex work, age at sexual debut, age at starting sex work, usual place of solicitation, sex clients volume per week, regular and occasional clients, ever having faced physical violence, ever receiving HIV test results, and condom use with regular partners were included in this study. 

## Analytical Approach

Bivariate analyses were carried out to understand the proportion of FSWs who were members of a CG by selected background characteristics. Chi-square statistics were applied to test the bivariate association in exposure to CG by selected background characteristics. Difference between means was assessed using the student T-test. All tests were double sided and a p-value of <0.05 was considered statistically significant. 

In order to examine the impact of CG exposure on the presence of any STI (laboratory tested), self-reported STI symptoms, and treatment-seeking behavior, radius caliper method of propensity score matching (PSM) [45,46] was applied. In this, each exposed participant was matched only with control participants whose propensity score fell in a predefined neighborhood of the estimated propensity score of the exposed. In the absence of experimental or case-control study/data, PSM method can be applied to assess the impact of exposure/treatment on program outcomes through observational study/data [47-52]. All selected variables, namely, age of participant, literacy status, source of income, current marital status, age at sexual debut, usual place of solicitation, and regular and occasional clients, were associated with CG and used to calculate the propensity score.

Propensity score is estimated by logistic regression, with the dichotomous outcome being the exposure/treatment (1 = exposed to CG; 0 = unexposed to CG) using associated observed background characteristics of the predictor variables. The key assumption in this method is that conditional on propensity score, assignment to the exposed (exposed to CG) and control group (unexposed to CG) can be taken as random [47]. To test this assumption, conditional on propensity score, the observable selected characteristics of the two groups have similar distributions. Even if this ‘balancing’ property is satisfied, we still have to assume that selection to the exposed group is not based on unobservable characteristics that also affect outcome variables. A better approach would be to match the observable characteristics measured before CG membership, since these should not be influenced by CG.

In this case, difference in STI presence between exposed and control groups can be directly compared to show the impact of exposure on the exposed group, known as average treatment effect on treated (ATT). Additionally, comparing the difference in STI presence between control and matched exposed groups can show the impact of exposure on the unexposed, known as average treatment effect on untreated (ATU). These two average effects were weighted by the proportion of FSWs in exposed and control groups, respectively, to arrive at the impact of the intervention exposure on presence of STIs, known as average treatment effect (ATE), which measured the increase/decrease in STIs due to CG exposure.

We first examined the impact of CG exposure on presence of any STI (laboratory tested), self-reported STI symptoms, and STI treatment-seeking behavior by comparing the presence of STIs and treatment-seeking behavior of FSWs exposed to CG against that among matched control FSWs. To assess whether the average effect is statistically significant, we estimated bootstrapped SE around the estimates [53,54]. Data was analyzed using STATA 11.0 [55].

## Results

### Exposure to CG and Socio-demographic Characteristics

The mean age of participants was 31.5 years. Of the 7,806 respondents, 46% were literate, 92% had ever been married, and more than three-fourth had sexual debut at the age of 15 years or later (77.6%), started sex work after the age of 20 years (84%), operated from a public place for solicitation (78.6%), and had regular (86.6%) and occasional (95.8%) clients.

A total of 2,939 (37.7%) FSWs reported being members of a CG. Members of CG were more likely to be aged above 24 years (86.4%), have ever been married (73%), have made sexual debut after the age of 14 years (72.8%), have started sex work after the age of 19 years (84%), operate from a public place for solicitation (81.5%), have regular (90.4%) and occasional (97.4%) clients, have ever received HIV test results (75.6%), exposed to program (62.8%), and self-report any STI treatment-seeking behavior (93.2%). Members of CG were less likely to have any STIs (4.5%). Condom use with regular partners was the same irrespective of exposure to CG, however, those ever experiencing physical violence (22.9%) were higher among exposed CG compared to unexposed FSWs (see Table 1). 

**Table 1:** Proportion of FSWs who were exposed/unexposed to community groups by selected background characteristics (India, IBBA, 2009–2010).

**Background variables**	**Total**	**Exposed to community groups (%)**	**Unexposed to community groups (%)**	**p value (Chi^2^ test statistics and Student T-test)**
N	7,806	2,939 (37.7%)	4,867 (62.3%)	
Age				
Mean age in years (SD)	31.5 (7.5)	32.2 (7.0)	31.0 (7.7)	<0.050 (5.5)
Less than 25 years	16.4	13.6	18.7	
25 years or more	83.6	86.4	81.3	<0.001 (36.7)
Literacy				
Illiterate	53.7	50.7	56.1	
Literate	46.3	49.3	43.9	0.041 (22.7)
Source of income other than sex work				
Yes	55.2	58.9	52.2	
No	44.8	41.1	47.8	0.010 (34.1)
Current marital status				
Never married	8.2	4.8	10.9	
Ever married	91.8	73.0	69.6	<0.001 (98.1)
Age at sexual debut				
Below 15 years	22.4	27.2	18.6	
15 years or more	77.6	72.8	81.4	<0.001 (83.4)
Age at starting sex work				
Below 20 years	16.0	16.0	16.0	
20 years and above	84.0	84.0	84.0	0.999 (0.0)
Usual place of solicitation				
Public	78.6	81.5	76.3	
Non-public	21.4	18.5	23.7	<0.001 (31.0)
Sex client volume per week				
Less than 10	62.9	56.9	67.7	
10 or more	37.1	43.1	32.3	<0.001 (95.6)
Regular clients				
Yes	86.6	90.4	83.6	
No	13.4	9.6	16.4	<0.001 (76.0)
Occasional clients				
Yes	95.8	97.4	94.4	
No	4.2	2.6	5.6	<0.001 (41.6)
Ever had physical violence				
Yes	13.2	22.9	13.3	
No	86.7	77.1	86.7	<0.001 (123.7)
Ever received HIV test results				
Yes	67.3	75.6	60.6	
No	32.7	24.4	39.4	<0.001 (197.4)
Condom use with regular partner				
Yes	13.7	14.0	13.4	
No	86.3	86.0	86.6	0.820 (0.5)
Exposure to program				
Yes	57.2	62.8	53.5	
No	42.8	38.2	46.5	<0.002 (54.4)
Presence of any STI (NG/CT/RPRTITER/TPHA)				
Yes	6.5	4.5	8.2	
No	93.5	95.5	91.8	<0.001 (43.7)
Self-reported any STI symptoms				
Yes	31.4	68.2	68.4	
No	68.6	31.8	31.6	0.261 (8.1)
STI treatment-seeking behavior *				
Yes	86.5	93.2	81.7	
No	13.5	6.8	18.3	<0.001 (68.9)

* Only those who self-reported any STI symptoms

### Impact of CG Exposure on Selected Outcomes

The impact of CG exposure on the presence of any STI, self-reported STI symptoms, and treatment-seeking behavior in matched samples of exposed and control groups is presented in Table 2. The average effect of CG exposure is the difference in presence of any STI and treatment-seeking behavior between exposed and control groups in outcome variables. Overall, balance properties were achieved for all statistically significant impact, confirming no significant difference in covariate between exposed and control groups, and hence that the ATT, ATU and ATE estimates obtained from the model were consistent.

**Table 2:** Estimated impact of exposure to community groups on STI-related outcomes among female sex workers.

**Outcome variables**	**Treated^#^ (%)**	**Untreated^$^ (%)**	**ATT (%)**	**ATU (%)**	**ATE (%)**	**CI (%)**
Presence of any STI	5.4	9.6	(-)4.2	(-)3.8	(-)4.0	(-)5.6 – (-)3.4
Self-reported any STI symptoms	31.8	32.1	(-)0.3	1.0	0.5	(-)1.8 - 2.2
STI treatment-seeking behavior	92.8	78.9	13.8	13.6	13.7	12.0 - 17.1

^#^ Treated (exposed to community group membership)

^$^ Untreated (Unexposed to community group membership)

ATT- Average treatment effect among treated

ATU- Average treatment effect among untreated

ATE- Average treatment effect

Exposure to CG helps in lowering STI presence by 4% [95% confidence interval (CI): -5.6 - -3.4, p<0.001] as compared to no exposure. However, this was not found in self-reporting of any STI symptoms. When compared to no exposure, CG exposure increased self-reported STI symptom treatment-seeking behavior by 13.7% (95% CI: 12.0-17.1, p<0.001). 

## Discussion

Our study of FSWs in the three Indian states of Andhra Pradesh, Maharashtra and Tamil Nadu found more than one third FSWs to be members of a CG. Most FSWs with membership to a CG were aged above 24 years, had ever been married, operated from a public place for solicitation, and had ever received HIV test results. FSWs with exposure to a CG reported significantly lower STI prevalence, compared to those who were not exposed. CG exposure did not have a statistically significant impact on self-reported STI symptoms. CG membership, compared to no such membership, had a greater impact on self-reported STI treatment-seeking behavior.

Most notably, we found that CG exposure resulted in decreased STI prevalence among FSWs. It substantiates that HIV education interventions undertaken through community mobilization help in declining STI prevalence among FSWs in India [22,26] and other developed/developing countries [56,57]. Earlier research shows that organizing FSWs into support groups and community-based organizations can help the community in collectively challenging structural barriers that increase their vulnerability to HIV/STI, including stigma, discrimination and social inequity [16,25]. Information on CGs is the most important package of services to increase condom use and reduce the risk of STI and HIV in India [23,32,43]. 

However, our study findings indicate that CG membership did not have an impact on self-reported STI symptoms. One possible reason for this statistically insignificant impact on self-reported STI symptoms could be the recall bias due to STI symptoms being non-specific and the fact that discharge could be from non-STI causes as well. Another important finding of our study was that CG membership increased the reported STI treatment from authorized health centers. Many studies validate this finding that STI prevention information provided by a CG peer educator on seeking STI treatment from health facilities appears to be most effective package of services to increase self-reported STI treatment-seeking behavior among FSWs [58-61]. 

Current research findings reiterate that community mobilization is an approach to improve health and treatment-seeking behavior among FSWs in targeted interventions. Such community based interventions could include strategies to create a sustainable condition that is empowering for FSWs [43,62], ensures safe sexual practices with different partners [23,24] and promote STI treatment-seeking behavior from public and private health facilities-supported STI treatment centers [33,43]. Therefore, efforts to mobilize, motivate and build the capacity of FSWs and their CGs are likely to improve the health of FSWs and could be strengthened with specific interventions in India.

 Although findings of this study offer important insights into bio-behavioral outcomes and their association with CG exposure, these results must be interpreted in the light of study limitations. First, self-reported STI symptoms may be affected by recall bias and be underreported in the survey. Second, the information on duration since associated/member of/with community group was not available and therefore has not been accounted for in the current analysis. . Third, estimates from PSM rely on conditional independence assumption, that is, all available variables considered to be predictor of exposure to CG are included in the model used to estimate propensity scores. However it is possible that other potential predictors of exposure to CG such as income and duration of STI and membership in community group were not available from our study and therefore could not be accounted in the model. . Finally, findings are specific to FSWs from the selected three high-HIV prevalence states of India. They cannot be generalized to other FSWs in the country.

The present study findings offer additional evidence that FSWs exposed to community groups have improved STI treatment behavior as compared to non-members. However, reporting of membership in community groups needs to be further improved and additional interventions and operation research are needed to build upon the evidences found in our study. Globally, community mobilization as a structural intervention approach- is considered critical to HIV prevention, and it has been recognized that such interventions are more effective for increased community participation and achieving HIV risk reduction outcomes [14,22,23,39,40]. And, data findings highlight the need for renewed attention to community mobilization in Andhra Pradesh, Maharashtra and Tamil Nadu state of India, with focus on improved access to STI testing and treatment and FSW-tailored counseling. 

## References

[1] Beeker C, Guenther-Grey C, Raj A (1998) Community empowerment paradigm drift and the primary prevention of HIV/AIDS. Soc Sci Med 46: 831-842. doi:10.1016/S0277-9536(97)00208-6. PubMed: 9541069. 9541069

[2] Evans C, Lambert H (2008) Implementing community interventions for HIV prevention: insights from project ethnography. 10.1016/j.socscimed.2007.08.030 17920740

[3] Pinto RM, McKay MM, Escobar C (2008) "You've gotta know the community": minority women make recommendations about community-focused health research. Women Health 47: 83-104. doi:10.1300/J013v47n01_05. PubMed: 18581694. 18581694 PMC2666258

[4] Galavotti C, Wheeler T, Kuhlmann AS, Saggurti N, Narayanan P et al. (2012) Navigating the swampy lowland: a framework for evaluating the effect of community mobilisation in female sex workers in Avahan, the India AIDS Initiative. J Epidemiol Community Health 66: ii9-ii15. doi:10.1136/jech-2011-200465. PubMed: 22760219. 22760219 PMC3603680

[5] Rau B (2006) The politics of civil society in confronting HIV/AIDS. Int Aff 82: 285-295. doi:10.1111/j.1468-2346.2006.00531.x.

[6] Chattopadhyay A, McKaig RG (2004) Social development of commercial sex workers in India: an essential step in HIV/AIDS prevention. AIDS Patient Care STDs 18: 159-168. doi:10.1089/108729104322994847. PubMed: 15104876. 15104876

[7] Herek GM (1999) AIDS and Stigma. Am Behav Sci 42: 1106-1116. doi:10.1177/00027649921954787.

[8] Parker R, Aggleton P (2003) HIV and AIDS-related stigma and discrimination: a conceptual framework and implications for action. Soc Sci Med 57: 13-24. doi:10.1016/S0277-9536(02)00304-0. PubMed: 12753813. 12753813

[9] Valdiserri RO (2002) HIV/AIDS stigma: an impediment to public health. Am J Public Health 92: 341-342. doi:10.2105/AJPH.92.3.341. PubMed: 11867303. 11867303 PMC1447072

[10] Parker R (2002) The global HIV/AIDS pandemic, structural inequalities, and the politics of international health. Am J Public Health 92: 343-346. doi:10.2105/AJPH.92.3.343. PubMed: 11867304. 11867304 PMC1447073

[11] Blankenship KM, Friedman SR, Dworkin S, Mantell JE (2006) Structural interventions: concepts, challenges and opportunities for research. J Urban Health 83: 59-72. doi:10.1007/s11524-005-9007-4. PubMed: 16736355. 16736355 PMC1473169

[12] Campbell C, Mzaidume Z (2001) Grassroots participation, peer education, and HIV prevention by sex workers in South Africa. Am J Public Health 91: 1978-1986. doi:10.2105/AJPH.91.12.1978. PubMed: 11726380. 11726380 PMC1446919

[13] Cornish F, Campbell C (2009) The social conditions for successful peer education: a comparison of two HIV prevention programs run by sex workers in India and South Africa. Am J Community Psychol 44: 123-135. doi:10.1007/s10464-009-9254-8. PubMed: 19521765. 19521765

[14] Cornish F, Ghosh R (2007) The necessary contradictions of 'community-led' health promotion: a case study of HIV prevention in an Indian red light district. Soc Sci Med 64: 496-507. doi:10.1016/j.socscimed.2006.09.009. PubMed: 17055635. 17055635

[15] Shahmanesh M, Patel V, Mabey D, Cowan F (2008) Effectiveness of interventions for the prevention of HIV and other sexually transmitted infections in female sex workers in resource poor setting: a systematic review. Trop Med Int Health 13: 659-679. doi:10.1111/j.1365-3156.2008.02040.x. PubMed: 18266784. 18266784

[16] Ghose T, Swendeman D, George S, Chowdhury D (2008) Mobilizing collective identity to reduce HIV risk among sex workers in Sonagachi, India: The boundaries, consciousness, negotiation framework. Social Science &amp. Medicine 67: 311-320. 10.1016/j.socscimed.2008.03.045 PMC2881846 18455855

[17] Biradavolu MR, Burris S, George A, Jena A, Blankenship KM (2009) Can sex workers regulate police? Learning from an HIV prevention project for sex workers in southern India. Soc Sci Med 68: 1541-1547. doi:10.1016/j.socscimed.2009.01.040. PubMed: 19261364. 19261364

[18] Campbell C, Nair Y, Maimane S (2007) Building contexts that support effective community responses to HIV/AIDS: a South African case study. Am J Community Psychol 39: 347-363. doi:10.1007/s10464-007-9116-1. PubMed: 17447133. 17447133

[19] Argento E, Reza-Paul S, Lorway R, Jain J, Bhagya M et al. (2011) Confronting structural violence in sex work: lessons from a community-led HIV prevention project in Mysore, India. AIDS Care 23: 69-74. doi:10.1080/09540121.2010.498868. PubMed: 21218278. 21218278

[20] Benzaken AS, Galbán Garcia E, Sardinha JC, Pedrosa VL, Paiva V (2007) [Community-based intervention to control STD/AIDS in the Amazon region, Brazil]. Rev Saude Publica 41 Suppl 2: 118-126. doi:10.1590/S0034-89102007000900018. PubMed: 18094795. 18094795

[21] Busza J, Baker S (2004) Protection and participation: an interactive programme introducing the female condom to migrant sex workers in Cambodia. AIDS Care 16: 507-518. doi:10.1080/09540120410001683457. PubMed: 15203418. 15203418

[22] Halli SS, Ramesh BM, O'Neil J, Moses S, Blanchard JF (2006) The role of collectives in STI and HIV/AIDS prevention among female sex workers in Karnataka, India. AIDS Care 18: 739-749. doi:10.1080/09540120500466937. PubMed: 16971283. 16971283

[23] Blankenship KM, West BS, Kershaw TS, Biradavolu MR (2008) Power, community mobilization, and condom use practices among female sex workers in Andhra Pradesh, India. AIDS 22 Suppl 5: S109-S116. doi:10.1097/01.aids.0000343769.92949.dd. PubMed: 19098471. 19098471

[24] Basu I, Jana S, Rotheram-Borus MJ, Swendeman D, Lee SJ et al. (2004) HIV prevention among sex workers in India. J Acquir Immune Defic Syndr 36: 845-852. doi:10.1097/00126334-200407010-00012. PubMed: 15213569. 15213569 PMC2826108

[25] Swendeman D, Basu I, Das S, Jana S, Rotheram-Borus MJ (2009) Empowering sex workers in India to reduce vulnerability to HIV and sexually transmitted diseases. Soc Sci Med 69: 1157-1166. doi:10.1016/j.socscimed.2009.07.035. PubMed: 19716639. 19716639 PMC2824563

[26] Reza-Paul S, Beattie T, Syed HU, Venukumar KT, Venugopal MS et al. (2008) Declines in risk behaviour and sexually transmitted infection prevalence following a community-led HIV preventive intervention among female sex workers in Mysore, India. AIDS 22 Suppl 5: S91-100. doi:10.1097/01.aids.0000343767.08197.18. PubMed: 19098483. 19098483

[27] Ramesh BM, Beattie TS, Shajy I, Washington R, Jagannathan L et al. (2010) Changes in risk behaviours and prevalence of sexually transmitted infections following HIV preventive interventions among female sex workers in five districts in Karnataka state, south India. Sex Transm Infect 86 Suppl 1: i17-i24. doi:10.1136/sti.2009.038513. PubMed: 20167725. 20167725 PMC3252604

[28] Jana S, Basu I, Rotheram-Borus MJ, Newman PA (2004) The Sonagachi Project: a sustainable community intervention program. AIDS Educ Prev 16: 405-414. doi:10.1521/aeap.16.5.405.48734. PubMed: 15491952. 15491952

[29] Sarkar S (2010) Community engagement in HIV prevention in Asia: going from ‘for the community’ to ‘by the community’—must we wait for more evidence? Sex Transm Infect 86: i2-i3. doi:10.1136/sti.2009.039289. PubMed: 20167726. 20167726 PMC3252600

[30] Biradavolu MR, Blankenship KM, Jena A, Dhungana N (2012) Structural stigma, sex work and HIV: contradictions and lessons learnt from a community-led structural intervention in southern India. J Epidemiol Community Health 66: ii95-ii99. doi:10.1136/jech-2011-200508. PubMed: 22705653. 22705653

[31] Foundation BMG (2008) Avahan—The India AIDS Initiative: The business of HIV prevention at scale. New Delhi: Bill & Melinda Gates Foundation.

[32] Chandrasekaran P, Dallabetta G, Loo V, Mills S, Saidel T et al. (2008) Evaluation design for large-scale HIV prevention programmes: the case of Avahan, the India AIDS initiative. AIDS 22 Suppl 5: S1-15. doi:10.1097/QAD.0b013e3282f4243b. PubMed: 19098469. 19098469

[33] Parimi P, Mishra RM, Tucker S, Saggurti N (2012) Mobilising community collectivisation among female sex workers to promote STI service utilisation from the government healthcare system in Andhra Pradesh, India. J Epidemiol Community Health 66: ii62-ii68. doi:10.1136/jech-2011-200832. PubMed: 22493478. 22493478

[34] Gaikwad SS, Bhende A, Nidhi G, Saggurti N, Ranebennur V (2012) How effective is community mobilisation in HIV prevention among highly diverse sex workers in urban settings? The Aastha intervention experience in Mumbai and Thane districts, India. J Epidemiol Community Health 66: ii69-ii77. doi:10.1136/jech-2011-200514. PubMed: 22760223. 22760223

[35] Punyam S, Pullikalu RS, Mishra RM, Sandri P, Mutupuru BP et al. (2012) Community advocacy groups as a means to address the social environment of female sex workers: a case study in Andhra Pradesh, India. J Epidemiol Community Health 66: ii87-ii94. doi:10.1136/jech-2011-200478. PubMed: 22495774. 22495774 PMC3433220

[36] Chakravarthy JBR, Joseph SV, Pelto P, Kovvali D (2012) Community mobilisation programme for female sex workers in coastal Andhra Pradesh, India: processes and their effects. J Epidemiol Community Health 66: ii78-ii86. doi:10.1136/jech-2011-200487. PubMed: 22945909. 22945909

[37] Narayanan P, Moulasha K, Wheeler T, Baer J, Bharadwaj S et al. (2012) Monitoring community mobilisation and organisational capacity among high-risk groups in a large-scale HIV prevention programme in India: selected findings using a Community Ownership and Preparedness Index. J Epidemiol Community Health 66: ii34-ii41. doi:10.1136/jech-2012-201065. PubMed: 22766780. 22766780

[38] Erausquin JT, Biradavolu M, Reed E, Burroway R, Blankenship KM (2012) Trends in condom use among female sex workers in Andhra Pradesh, India: the impact of a community mobilisation intervention. J Epidemiol Community Health 66: ii49-ii54. doi:10.1136/jech-2011-200511. PubMed: 22495773. 22495773 PMC3433222

[39] Guha M, Baschieri A, Bharat S, Bhatnagar T, Sane SS et al. (2012) Risk reduction and perceived collective efficacy and community support among female sex workers in Tamil Nadu and Maharashtra, India: the importance of context. J Epidemiol Community Health 66: ii55-ii61. doi:10.1136/jech-2011-200562. PubMed: 22760217. 22760217

[40] Saggurti N, Mishra RM, Proddutoor L, Tucker S, Kovvali D et al. (2013) Community collectivization and its association with consistent condom use and STI treatment-seeking behaviors among female sex workers and high-risk men who have sex with men/transgenders in Andhra Pradesh, India. AIDS Care 25: S55-S66. doi:10.1080/09540121.2012.749334. PubMed: 23745631. 23745631 PMC4003583

[41] Bhattacharjee P, Prakash R, Pillai P, Isac S, Haranahalli M et al. (2013) Understanding the role of peer group membership in reducing HIV-related risk and vulnerability among female sex workers in Karnataka, India. AIDS Care 25: S46-S54. doi:10.1080/09540121.2012.736607. PubMed: 23745630. 23745630 PMC4003574

[42] ICMR, FHI360 (2011) Integrated Behavioural and Biological Assessment (IBBA): Guidelines for Survyes of Population at Risk of HIV Infection. New Delhi Indian; Council of Medical Research and FHI360 256 p

[43] BMGF (2008) Avahan, the India AIDS Initiative- the business of HIV prevention at scale. New Delhi: Bill & Melinda Gates Foundation.

[44] Saidel T, Adhikary R, Mainkar M, Dale J, Loo V et al. (2008) Baseline integrated behavioural and biological assessment among most at-risk populations in six high-prevalence states of India: design and implementation challenges. AIDS 22 Suppl 5: S17-S34. PubMed: 19098477. 10.1097/01.aids.0000343761.77702.04 19098477

[45] Rosenbaum PR, Rubin DB (1985) Constructing a Control Group Using Multivariate Matched Sampling Methods That Incorporate the Propensity Score. Am Stat 39: 33-38.

[46] Stuart EA (2010) Matching methods for causal inference: A review and a look forward. Stat Sci 25: 1-21. PubMed: 20871802. 20871802 10.1214/09-STS313 PMC2943670

[47] Rosenbaum PR, Rubin DB (1983) The central role of the propensity score in observational studies for causal effects. Biometrika 70: 41-55.

[48] Rubin DB, Thomas N (1996) Matching using estimated propensity scores: relating theory to practice. Biometrics 52: 249-264. PubMed: 8934595. 8934595

[49] Williamson E, Morley R, Lucas A, Carpenter J (2012) Propensity scores: from naive enthusiasm to intuitive understanding. Stat Methods Med Res 21: 273-293. PubMed: 21262780. 21262780 10.1177/0962280210394483

[50] Mahal A, Canning D, Odumosu K, Okonkwo P (2008) Assessing the economic impact of HIV/AIDS on Nigerian households: a propensity score matching approach. AIDS 22 Suppl 1: S95-101. PubMed: 18664961. 10.1097/01.aids.0000327629.62350.59 18664961

[51] Dehejia RH, Wahba S (2002) Propensity Score-Matching Methods for Nonexperimental Causal Studies. Rev Econ Statist 84: 151-161.

[52] Juneja S, Rao Tirumalasetti V, Mishra RM, Sethu S, Singh IR (2013) Impact of an HIV Prevention Intervention on Condom Use among Long Distance Truckers in India. AIDS Behav 17: 1040-1051. PubMed: 23008122. 23008122 10.1007/s10461-012-0314-y PMC3586141

[53] Lechner M (2002) Some practical issues in the evaluation of heterogeneous labour market programmes by matching methods. J R Stat Soc A (Statist Soc) 165: 59-82.

[54] Oakes JM, Kaufman JS, editors (2006) Methods in Social Epidemiology. San Francisco, CA: John Wiley & Sons.

[55] StataCorp (2009) Stata Statistical Software: Release 11 College Station, TX: StataCorp LP.

[56] Ford K, Wirawan DN, Reed BD, Muliawan P, Wolfe R (2002) The Bali STD/AIDS Study: evaluation of an intervention for sex workers. Sex Transm Dis 29: 50-58. PubMed: 11773879. 11773879 10.1097/00007435-200201000-00009

[57] Rou K, Wu Z, Sullivan SG, Li F, Guan J et al. (2007) A five-city trial of a behavioural intervention to reduce sexually transmitted disease/HIV risk among sex workers in China. AIDS 21 Suppl 8: S95-101. PubMed: 18172399. 10.1097/01.aids.0000304703.77755.c7 18172399

[58] NACO (2006) NACP-III - To Halt and Reverse the HIV Epidemic in India. New Delhi: Ministry of Health and Family Welfare, Government of India.

[59] Gunn RA, Rolfs RT, Greenspan JR, Seidman RL, Wasserheit JN (1998) The changing paradigm of sexually transmitted disease control in the era of managed health care. JAMA 279: 680-684. PubMed: 9496986. 9496986 10.1001/jama.279.9.680

[60] Mayaud P, McCormick D (2001) Interventions against sexually transmitted infections (STI) to prevent HIV infection. Br Med Bull 58: 129-153. PubMed: 11714628. 11714628 10.1093/bmb/58.1.129

[61] Kane S, Dewan PK, Gupta D, Wi T, Das A et al. (2010) Large-scale public-private partnership for improving TB-HIV services for high-risk groups in India. Int J Tuberc Lung Dis 14: 1066-1068. PubMed: 20626954. 20626954

[62] Beattie TS, Bhattacharjee P, Ramesh BM, Gurnani V, Anthony J et al. (2010) Violence against female sex workers in Karnataka state, south India: impact on health, and reductions in violence following an intervention program. BMC Public Health 10: 476 PubMed: 20701791. 20701791 10.1186/1471-2458-10-476 PMC2931467

